# Medical Therapy with Pasireotide in Recurrent Cushing’s Disease: Experience of Patients Treated for At Least 1 Year at a Single Center

**DOI:** 10.3389/fendo.2017.00035

**Published:** 2017-02-27

**Authors:** Chris G. Yedinak, Sarah Hopkins, Jessica Williams, Aly Ibrahim, Justin Schultz Cetas, Maria Fleseriu

**Affiliations:** ^1^Department of Medicine–Endocrinology, Diabetes, and Clinical Nutrition, OHSU Northwest Pituitary Center, Oregon Health & Science University, Portland, OR, USA; ^2^Department of Neurological Surgery, OHSU Northwest Pituitary Center, Oregon Health & Science University, Portland, OR, USA

**Keywords:** pasireotide, real-world clinical experience, hyperglycemia, Cushing’s disease, recurrent Cushing’s disease

## Abstract

Subcutaneous (SC) injection of pasireotide, a somatostatin analog, is approved for the treatment of adults with Cushing’s disease (CD) for whom pituitary surgery was unsuccessful or is not an option. We highlight the symptomatic and biochemical improvement of six patients with recurrent CD treated with pasireotide SC at a single center for at least 1 year. Patients were treated either through commercial use (*n* = 5) or through the Phase 3 trial (*n* = 1; http://ClinicalTrials.gov identifier, NCT00434148; study number, B2305). Most patients (*n* = 5) were female, and the mean age at diagnosis was 35.8 years. All patients demonstrated biochemical control at 1 year of treatment. Three of the five real-world patients followed for more than 1 year remain on pasireotide SC and are controlled. Two patients discontinued pasireotide SC; one patient because of persistently elevated urinary-free cortisol levels and gallstones, and the other because of treatment for an unrelated brain tumor. Symptomatic improvement varied, but all patients demonstrated weight loss. Nausea and mild, transient injection-site reactions were the most frequently reported adverse events. Although glycated hemoglobin (HbA_1c_) increased after treatment initiation, four of five patients maintained HbA_1c_ levels ≤7.0% while receiving pasireotide SC and concomitant individualized diabetes medication, if necessary. In patients who discontinued pasireotide SC, HbA_1c_ levels decreased within 6 weeks. This report documents real-world use of pasireotide SC and indicates its effectiveness as a long-term treatment option for patients with CD. Although hyperglycemia was observed in most patients, it was managed with appropriate monitoring and treatment and was reversible upon discontinuation of pasireotide SC.

## Background

Cushing’s disease (CD) has an estimated incidence of 6.2–7.6 per million person-years in the United States (1) and is the most common cause of Cushing’s syndrome, accounting for nearly 70% of cases (2). CD is characterized by an elevation in cortisol levels caused by an adrenocorticotropic hormone (ACTH)-secreting pituitary adenoma (3, 4). Failure to accurately identify and treat patients with the disease could lead to an increased risk of mortality and worsened morbidity compared with the control population. Diagnosis of CD may be challenging because many of the signs and symptoms of hypercortisolism are non-specific, and similar to common health conditions such as hypertension, impaired glucose tolerance (diabetes), obesity, and osteoporosis (3, 4).

While transsphenoidal surgical resection of the corticotroph tumor is the recommended first-line treatment for patients with CD (3–6), lifelong recurrence rates are 23–33% after initial remission (5). Results from a meta-analysis of 74 studies published between 1976 and 2014 showed that mean remission rates in patients with pituitary microadenomas and macroadenomas were 82.1% (median, 85.7%; range, 48.7–100%) and 62.3% (median, 64.1%; range, 30.8–100%), respectively (6). Mean recurrence rates reported in patients with microadenomas and macroadenomas were 11.7% (median, 10.9%; range, 0–36.4%) and 18.8% (median, 13.9%; range 0–59%), respectively (6). In addition, surgical failures, including disease persistence and recurrence after surgery, are more common in patients with macroadenomas (mean, 48.8%; median, 52.2%; range, 0–71.4%) than in patients with microadenomas (mean, 25%; median, 21.2%; range, 0–55.5%) (6). Thus, in cases of postsurgical disease recurrence or those in which surgery is not appropriate, medical therapy plays an important role in the long-term management of CD (5–7).

Pasireotide subcutaneous (SC) injection is a somatostatin analog approved for the treatment of adults with CD for whom pituitary surgery was unsuccessful or is not an option (7). In a Phase 3 trial in patients with CD (http://ClinicalTrials.gov identifier, NCT00434148; study number B2305), treatment with pasireotide SC was associated with normalization of urinary-free cortisol (UFC) levels at 6 months in 15% of patients treated with a 0.6-mg twice-daily (bid) dose and 26% of patients treated with a 0.9-mg bid dose (8). The B2305 study also demonstrated significant improvements in the signs and symptoms of CD and reductions in pituitary tumor volume. A separate analysis of the data from this Phase 3 trial showed marked improvements in the signs and symptoms of CD during 12 months of pasireotide treatment while UFC levels decreased (9).

Overall, there are few reports of long-term treatment with pasireotide SC. Data obtained in the open-ended, open-label extension trial of the B2305 study showed that the mean decrease in the UFC level after 12 months of treatment (57.3%) was maintained through 24 months (62.1%), suggesting that the efficacy and clinical benefit of pasireotide SC are sustained over a longer period of time (10). A single-center study in Italy reported that treatment with pasireotide led to a substantial reduction in tumor size within 6–12 months of initiation in a subset of eight patients from the B2305 study (11). In a 5-year follow-up of 16 patients who remained on treatment, UFC reductions and clinical improvements were found to be sustained (12).

Data on patients with CD in a non-research setting are limited. This article describes the symptomatic and biochemical responses of six patients with recurrent CD treated with pasireotide SC for at least 1 year at a single center, five of whom were treated in a real-world setting.

## Introduction

Five patients were treated with pasireotide SC for at least 1 year as part of standard clinical care at the Oregon Health & Science University, Northwest Pituitary Center, Portland, OR, USA. Chart review was performed to assess patient baseline characteristics, treatment course, symptomatic and biochemical responses, adverse events (AEs), and the tolerability of pasireotide SC over time. UFC assays were performed at a single diagnostic laboratory using liquid chromatography-tandem mass spectrometry (LC-MS/MS). All glycated hemoglobin (HbA_1c_) analyses were performed using an immunochemical assay; salivary cortisol assays were performed at the same laboratory using enzyme-linked immunosorbent methodology. One additional patient at this center was part of the B2305 study group, which was a Phase 3, double-blind, randomized, multicenter trial that evaluated the control of UFC by pasireotide SC in patients with confirmed persistent or recurrent CD after surgery, or those with newly diagnosed CD who were not candidates for surgery (http://ClinicalTrials.gov identifier, NCT00434148). The primary endpoint was normalization of UFC level. Other endpoints included changes in serum and salivary cortisol levels over time, changes in clinical signs and symptoms, quality of life, and safety. Patients were randomly assigned to receive pasireotide SC at doses of 0.6 or 0.9 mg bid. This study design has been previously described in detail by Colao et al. (8). The Oregon Health & Science University institutional review board (IRB) approved both the prospective and the retrospective study, which included these patients and data analysis for this study. Written informed consent from individual patients in the retrospective study was waived per IRB protocol. Written informed consent was obtained from the patient in the prospective clinical trial.

### Investigation

The mean age of the six study patients at diagnosis was 35.8 years, and most patients were female (*n* = 5; Table 1). The mean disease duration was 12 years (range, 3–25 years). The most common symptoms reported by patients included abnormal weight gain, fatigue, cushingoid face, and ecchymosis. All patients underwent surgery, and the median time to recurrence after surgery was approximately 3 years (range, 5 months–22 years). All patients had at least 1 elevated salivary cortisol value at the time of recurrence.

**Table 1:** **Patient information**.

	Patient 1	Patient 2	Patient 3	Patient 4	Patient 5	Patient 6
Sex	F	F	F	F	F	M
Age at diagnosis	45	35	38	31	45	21
MRI findings at diagnosis	Not available	7 mm	6 mm	Not available	Not available	7–8 mm
Postsurgical time to recurrence, months	60	5	9	264	33	30
Medical therapies prior or in addition to pasireotide SC	Cabergoline started simultaneously	None	Ketoconazole followed by mifepristone (intolerant to mifepristone)	Cabergoline without improvement	Ketoconazole (without control) followed by cabergoline (some improvement)	None (SOM230 clinical trial patient)
Pasireotide SC dose, mg/bid	0.3 → 0.9 → 0.6 → 0.3	0.3 → 0.6	0.6 → 0.9 → 0.6	0.6 → 0.9 → 0.6	0.3 → 0.6 → 0.3	0.6
UFC after treatment (ULN) [baseline UFC], μg/24 h	10 (45) [13,120]	Not measured^a^	35 (45) [6.3]^b^	10.9 (45) [20.2]	10 (45) [222.2]	34 (60) [62.2]
Time to treatment response with pasireotide (months)	3	11	12	4	17	9
Status of pasireotide SC treatment	Ongoing	Ongoing	Ongoing	Stopped because of new GBM diagnosis	Ongoing	Stopped because of lack of biochemical control, gallstones, and pathological fracture
Total duration of treatment with pasireotide SC, months	25	25	22	16	23	31
Time in remission on pasireotide SC, months	22	11	19	16	17	Unclear

*bid, twice daily; GBM, glioblastoma; MRI, magnetic resonance imaging; SC, subcutaneous; UFC, urinary-free cortisol; ULN, upper limit of normal*.

*^a^Late-night salivary cortisol levels normalized to 1.9 from 8.2 nmol/L at baseline (ULN, 4.3 nmol/L) in patient No. 2*.

*^b^Initial UFC and salivary cortisol measurements for patient No. 3 were low*.

*Diagnosis was confirmed based on symptoms and dexamethasone-suppressed corticotropin-releasing hormone stimulation test*.

### Treatment

Three patients were initially treated with pasireotide SC 0.6 mg bid, and three patients received an initial dose of 0.3 mg bid. Dosing was increased to 0.9 mg bid in three patients; dosing in these patients was subsequently reduced to 0.6 mg bid. The dose was further reduced to 0.3 mg bid in two patients. Two patients were naive to medical treatment (one of these patients was included in the B2305 study group), and the remaining four patients initiated pasireotide SC due to failure of or intolerance to other medical therapies (e.g., ketoconazole, mifepristone, cabergoline; Table 1). Pasireotide SC was used in combination with cabergoline (1.0–1.5 mg twice weekly) in two of the real-world setting patients. The mean duration of treatment in all cases was 23.6 months (range, 16–31 months). The average time to treatment response was 4 months.

### Outcome and Follow-up

**Figure 1:**
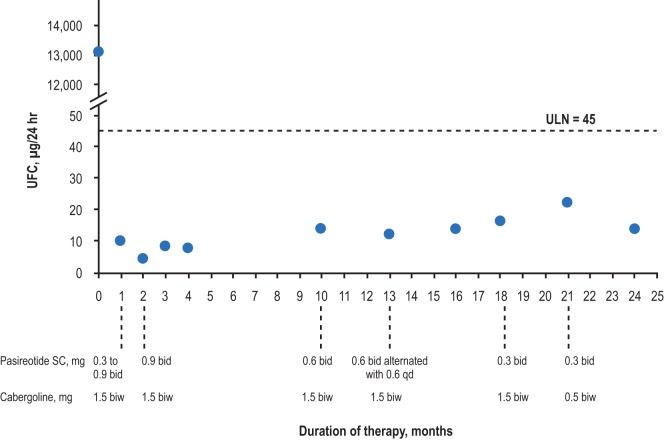
**Urinary-free cortisol levels of patient No. 1 over the duration of treatment**. bid, twice daily; biw, twice weekly; qd, once daily; UFC, urinary-free cortisol; ULN, upper limit of normal.

All patients demonstrated biochemical and clinical control at 1 year of treatment (e.g., Figure 1). Biochemical control was defined as normalization of either UFC or salivary cortisol based on current clinical guidelines and expert opinion publications. Four patients remain on pasireotide SC, three of whom are controlled. The remaining patient, who continues on 0.3 mg bid pasireotide SC therapy, had elevated salivary cortisol values shortly after 1 year of therapy, for which the dose was titrated up. Patients were monitored for 24-h UFC levels, serum cortisol, and ACTH, starting 1 month after the initiation of treatment and continuing every 3 months thereafter until cortisol levels normalized. The drug dose was titrated (with pasireotide SC uptitrated or downtitrated by 0.3 mg bid at each step, except for patient No. 1, for whom the pasireotide SC dose was initially uptitrated by 0.6 mg bid) to maintain cortisol levels and vital signs within normal ranges and avoid symptoms of adrenal insufficiency.

The patient treated in the B2305 study setting (patient No. 6) was controlled at the end of the study. However, patient No. 6 ultimately had recurrent UFC level elevations and gallstones that required discontinuation of pasireotide SC after 31 months of treatment. Another patient (patient No. 4) discontinued pasireotide SC treatment due to an unrelated brain tumor diagnosis and began high-dose dexamethasone treatment for that condition. The mean duration of treatment for patients continuing on the drug was 15 months (range, 12–17 months), with a mean time to normalization of UFC or late-night salivary cortisol levels of 6 months (range, 3–11 months). Specific symptomatic improvements varied across the six patients, but all patients demonstrated weight loss.

**Figure 2:**
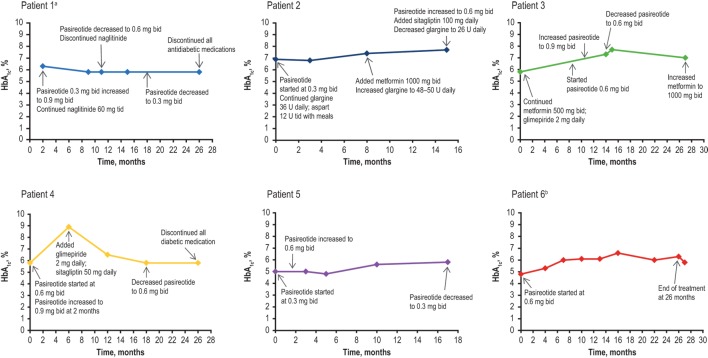
**HbA_1c_ levels of patients over the duration of treatment**. bid, twice daily; HbA_1c_, glycated hemoglobin; tid, three times daily. ^a^Baseline data not available. ^b^Metformin 500 mg bid was prescribed before the initial HbA_1c_ result and was stopped after 11 days when HbA_1c_ results showed low values.

All patients reported nausea, and three patients experienced injection-site reactions. Both AEs were considered to be mild in severity and transient. Nausea resolved with administration of injections after meals. Injection-site reactions were self-limiting; however, pretreatment with ibuprofen and the administration of ice to the site prior to injection were prescribed as initial treatment. The average baseline HbA_1c_ level was 5.9% (range, 4.8–7.5%), with increases ranging from 0.6 to 3.1% (Figure 2). Four out of the five real-world patients exhibited HbA_1c_ ≤7.0% at the last visit while receiving pasireotide SC. One of these four patients (patient No. 5) had a normal HbA_1c_ reading throughout the treatment and did not require antidiabetic medication. Patient No. 2 was diagnosed with diabetes mellitus before initiating treatment with pasireotide. Hyperglycemia was managed in all other cases by the provision of concomitant and individualized medical treatment, as necessary. Patient No. 1 received naglitinide 60 mg three times daily (tid), initially with 0.9 mg of pasireotide SC; naglitinide treatment was discontinued after approximately 8 months and the pasireotide SC dose was reduced to 0.6 mg. Similar discontinuation of diabetic medication was observed in patient No. 4, who received glimepiride 2 mg and sitagliptin 50 mg daily, initially with 0.6 mg of pasireotide SC. Patient No. 4’s HbA_1c_ increased from 5.8 to 8.9% after initiating pasireotide treatment. This patient was then administered antidiabetic treatment with glimepiride 2 mg daily and sitagliptin 50 mg daily, which reduced HbA_1c_ levels to 6.5%. Pasireotide SC was increased after 2 months to 0.9 mg. After 18 months, the pasireotide SC dose was reduced to 0.6 mg; after 26 months, the diabetic medications were discontinued. HbA_1c_ remained stable at 6.5% after discontinuation of antiglycemics. Patient No. 3 received metformin 500 mg bid and glimepiride 2 mg daily with pasireotide SC 0.6 mg. Metformin was increased to 1,000 mg bid to normalize HbA_1c_ levels after 28 months of treatment. Patient No. 2 received glargine 36 U daily and aspart 12 U tid, initially with 0.3 mg pasireotide SC. After 8 months, metformin 1,000 mg bid was initiated and glargine dosing was increased to 48–50 U daily. At month 15, the pasireotide SC dose was increased to 0.6 mg and sitagliptin 100 mg was taken daily; HbA_1c_ levels remained >7%, including after glargine was reduced to 26 U daily.

The B2305 study patient (patient No. 6) was initially treated with metformin 500 mg bid, which was reduced in 1 week to 500 mg daily, but treatment was discontinued 3 weeks after initial treatment when HbA_1c_ levels were observed to be low. All patients received counseling regarding diet and exercise. In general, increases in HbA_1c_ levels were reversible upon cessation of pasireotide SC.

## Discussion

This cross-sectional case series review provides the first real-world, long-term treatment evidence of the efficacy and safety of pasireotide SC as a viable medical treatment option for patients with recurrent CD. This chart review showed biochemical control and overall symptomatic improvement exhibited by all patients that varied over the course of long-term treatment. It also supports previous studies in the literature that highlight the importance of an early diagnosis of recurrence, thus improving the chances of control; elevated late-night salivary cortisol levels are an early biochemical marker of recurrent CD (5, 13, 14). Most patients experienced hyperglycemia, which was controlled by prescribed concomitant antidiabetic medication and periodic monitoring of the associated changes. Two cases of pasireotide SC discontinuation were neither immediate, nor directly related to treatment.

This case series provides helpful examples of the successful management of AEs in a clinical setting. Similar to other somatostatin analogs and previous clinical studies, the most common AEs experienced by patients were gastrointestinal and were managed by dietary modifications and the intermittent use of antidiarrheal agents, such as loperamide. Results from a mechanistic study conducted in healthy volunteers showed that pasireotide, independent of dosing, caused significant decreases in insulin levels without changes in hepatic or peripheral insulin sensitivity (15). In all of the cases presented here, hyperglycemia was promptly diagnosed during the early stages of treatment based on previous studies and guidelines for CD treatment that emphasize the monitoring of blood glucose and HbA_1c_ levels (16). Blood glucose levels were adequately controlled in accordance with the recommendations provided in the literature, which include following dietary restrictions, and individualized medical treatment regimens (16). Hyperglycemic effects were reported to be reversed within 6 weeks to 4 months of drug discontinuation, similar to other cases in the literature (17). Thus, these case studies highlight in a real-world setting the overall safety, tolerability, and manageability associated with pasireotide SC treatment that has been demonstrated in clinical trials.

These cases provide some indication of how pasireotide SC might perform in a broader patient population. They also provide an expanded understanding of drug interactions and outcomes under different scenarios, which is outside of the scope of clinical trial protocols. However, this study has certain limitations that should be considered when analyzing the impact of the outcomes. The analysis is retrospective and based on a limited number of patients compared with the large sample sizes of clinical trials. Another limitation to be noted is that biochemical control in patient No. 1 and patient No. 5 was achieved with a combination of pasireotide and cabergoline. Patient No. 1 began treatment with cabergoline 3 weeks before initiating pasireotide, and this combination therapy was continued. Thus, the concomitant treatment may have influenced UFC measurements and/or normalization. In addition, the data were obtained from a single center, whose treatment practices may not be reflective of those practiced by all clinics that treat patients with CD. Overall, these data help bridge the gap in knowledge between outcomes from well-defined and regulated clinical trials and actual clinical practice, resulting in broader understanding by physicians/prescribers of the potential medical impact of pasireotide SC.

## Concluding Remarks

This study addresses an unmet need for data on treatment patterns with pasireotide SC in a real-world setting in patients with recurrent CD. Safety outcomes and manageability were consistent with previous observations in clinical studies. In addition, this is the first study to describe successful hyperglycemia management with concomitant medication and adequate monitoring in a real-world practice. Correlations between dose adjustments in diabetic medications and pasireotide SC were also documented in this study. The evidence generated through these cases may assist in optimizing patient access and influencing treatment decisions by physicians, such as selecting patients who will benefit the most from this treatment.

## Ethics Statement

The study has been carried out in accordance with guidelines recommended by and it was approved by the OHSU IRB. The patient in the prospective clinical trial signed informed consent and the requirement for the other patients informed consent has been waived by the OHSU IRB.

## Author Contributions

CY, SH, and MF contributed to design the study, participated in data interpretation and drafting, and revised the manuscript. JW, AI, and JC participated in data interpretation and drafting and revised the manuscript. All authors read and approved the final version of the manuscript for publication.

## Conflict of Interest Statement

CY has served as a consultant for Chiasma, Ipsen, Novartis, and Pfizer. SH is a coinvestigator in clinical trials sponsored by Chiasma, Cortendo, and Novartis, with research support provided to Oregon Health & Science University. MF has been a principal investigator in clinical trials sponsored by Chiasma, Cortendo, Ipsen, Novartis, and Pfizer, with research support provided to Oregon Health & Science University. She has also served as an *ad hoc* consultant for Chiasma, Cortendo, Novartis, and Pfizer. AI has a fellowship partially funded by DePuy Synthes. JW and JC have nothing to disclose.
